# The Networks of Genes Encoding Palmitoylated Proteins in Axonal and Synaptic Compartments Are Affected in PPT1 Overexpressing Neuronal-Like Cells

**DOI:** 10.3389/fnmol.2017.00266

**Published:** 2017-08-22

**Authors:** Francesco Pezzini, Marzia Bianchi, Salvatore Benfatto, Francesca Griggio, Stefano Doccini, Rosalba Carrozzo, Arvydas Dapkunas, Massimo Delledonne, Filippo M. Santorelli, Maciej M. Lalowski, Alessandro Simonati

**Affiliations:** ^1^Neurology (Neuropathology and Child Neurology), Department of Neuroscience, Biomedicine and Movement, University of Verona Verona, Italy; ^2^Unit of Muscular and Neurodegenerative Disorders, IRCCS Bambino Gesù Children’s Hospital Rome, Italy; ^3^Functional Genomics Center, Department of Biotechnology, University of Verona Verona, Italy; ^4^Molecular Medicine, IRCCS Stella Maris Calambrone-Pisa, Italy; ^5^Medicum, Biochemistry/Developmental Biology, Meilahti Clinical Proteomics Core Facility, University of Helsinki Helsinki, Finland

**Keywords:** CLN1 disease, PPT1, differentiated neuroblastoma cells, RNA-seq, neuritogenesis, dysregulated genes, palmitoylation

## Abstract

CLN1 disease (OMIM #256730) is an early childhood ceroid-lipofuscinosis associated with mutated *CLN1*, whose product Palmitoyl-Protein Thioesterase 1 (PPT1) is a lysosomal enzyme involved in the removal of palmitate residues from S-acylated proteins. In neurons, PPT1 expression is also linked to synaptic compartments. The aim of this study was to unravel molecular signatures connected to *CLN1*. We utilized SH-SY5Y neuroblastoma cells overexpressing wild type *CLN1* (SH-p.wtCLN1) and five selected CLN1 patients’ mutations. The cellular distribution of wtPPT1 was consistent with regular processing of endogenous protein, partially detected inside Lysosomal Associated Membrane Protein 2 (LAMP2) positive vesicles, while the mutants displayed more diffuse cytoplasmic pattern. Transcriptomic profiling revealed 802 differentially expressed genes (DEGs) in SH-p.wtCLN1 (as compared to empty-vector transfected cells), whereas the number of DEGs detected in the two mutants (p.L222P and p.M57Nfs*45) was significantly lower. Bioinformatic scrutiny linked DEGs with neurite formation and neuronal transmission. Specifically, neuritogenesis and proliferation of neuronal processes were predicted to be hampered in the wt*CLN1* overexpressing cell line, and these findings were corroborated by morphological investigations. Palmitoylation survey identified 113 palmitoylated protein-encoding genes in SH-p.wtCLN1, including 25 ones simultaneously assigned to axonal growth and synaptic compartments. A remarkable decrease in the expression of palmitoylated proteins, functionally related to axonal elongation (GAP43, CRMP1 and NEFM) and of the synaptic marker SNAP25, specifically in SH-p.wtCLN1 cells was confirmed by immunoblotting. Subsequent, bioinformatic network survey of DEGs assigned to the synaptic annotations linked 81 DEGs, including 23 ones encoding for palmitoylated proteins. Results obtained in this experimental setting outlined two affected functional modules (connected to the axonal and synaptic compartments), which can be associated with an altered gene dosage of wt*CLN1*. Moreover, these modules were interrelated with the pathological effects associated with loss of PPT1 function, similarly as observed in the *Ppt1* knockout mice and patients with CLN1 disease.

## Introduction

Neuronal ceroid lipofuscinoses (NCL) are a group of lysosomal storage diseases (LSD) mainly affecting brain and retina (Mole et al., 2011). NCL are named after the unifying pathological hallmark, i.e., the accumulation of autofluorescent lipopigments with characteristic ultrastructural features in the cytoplasm of neurons and other cell types (Goebel et al., 1974; Haltia, 2003). Thirteen NCL genes have been identified so far and an equivalent number of NCL forms are currently considered in NCL nosography^1^. Five NCL genes encode lysosomal proteins, whereas the remaining gene products localize to other cell compartments. A definitive function has been established for a number of proteins only (Cárcel-Trullols et al., 2015). CLN1 disease (OMIM #256730) refers to a NCL form associated with a childhood onset phenotype (Santavuori et al., 1974; Vesa et al., 1995; Simonati et al., 2009), and a rare adult onset one (van Diggelen et al., 2001). Common biomarker of both phenotypes is the reduced enzyme activity (EA) of Palmitoyl-Protein Thioesterase 1 (PPT1), the gene product of *CLN1*, whose mutations are disease-related.

PPT1 is a palmitoyl-thioesterase highly expressed in neuronal cells. It is a soluble, hydrolytic lysosomal enzyme, involved in the degradation of S-acylated proteins by removing the palmitate residues (Hellsten et al., 1996; Verkruyse and Hofmann, 1996; Bellizzi et al., 2000). In neurons, it is also localized in other cellular compartments (such as axons and specialized neural endings) where its enzymatic activity is conveyed at neutral pH (Cho et al., 2000b; Shyng et al., 2017). This localization as well as the fact that its expression is developmentally regulated in both humans and mice is consistent with a neuron-specific function for PPT1 (Isosomppi et al., 1999; Heinonen et al., 2000). Several lines of evidences indicate that in nerve terminals PPT1 is involved in regulation of exo/endocytosis, synaptic vesicle recycling and eventually neurotransmission (Kim et al., 2008; Aby et al., 2013). Palmitoylation, the lipidic post-translational modification in which PPT1 is involved, regulates intracellular trafficking and shuttling of proteins among different membrane organelles, particularly in neuronal cells (Fukata and Fukata, 2010). Therefore, it is not surprising that several human disorders affecting neural functions, including schizophrenia, Huntington’s and CLN1 diseases are associated with defective palmitoylation (Hornemann, 2015). Although more than 20 enzymes promoting palmitoylation (Palmitoyl-Acyl Transferases, PATs, also known as zinc finger S-acyl transferases, zDHHC) are recognized, only three de-palmitoylating enzymes including PPT1 have been thus far identified (Fukata and Fukata, 2010; Blanc et al., 2015).

Both *in vivo* models (including zebrafish and mice) and *in vitro* systems have been extensively used to dissect out the role of PPT1 in neuronal tissues (Lyly et al., 2007; Bond et al., 2013; Faller et al., 2015). Among cellular models, neuroblastoma cells have been acknowledged as a useful system to investigate the effects of NCL genes expression on neuronal functions and protein interactions. Overexpression of *CLN1* protects LAN-5 neuroblastoma cells from ceramide-induced apoptosis (Cho and Dawson, 2000), whereas antisense treatment (leading to a reduced expression of PPT1) increased the susceptibility to undergo cell death (Cho et al., 2000a). Recently, SH-SY5Y neuroblastoma cells overexpressing either *CLN1, CLN3* or *CLN5* have been also used to recognize putative interacting proteins of PPT1 and CLN3/CLN5 crosstalk by proteomics approach (Scifo et al., 2013, 2015a,b).

The aim of this study was to recognize molecular signatures and functional modules connected with overexpressed *CLN1* in human neuronal cellular system. We utilized SH-SY5Y neuroblastoma cells (differentiated into a neuronal-like phenotype, Pezzini et al., 2017) to overexpress wt*CLN1* and a selection of disease related mutations previously detected in CLN1-affected children. The differentiated cell lines underwent whole transcriptomic profiling by RNA-seq, to identify differentially expressed genes (DEGs) which are functionally related to the overexpression of wild-type or mutated PPT1. Following bioinformatic investigations, we focused on DEGs involved in palmitoylation of neuronal proteins as well as related cellular functions. Interestingly, genes coding for palmitoylated proteins assigned to neuronal functions, such as axonal growth, and to the synaptic compartment were the most significantly expressed. Moreover, to identify potential therapeutic targets for CLN1 disease, we aimed to demonstrate possible links with other NCL genes, particularly *CLN4* and *CLN10*, sharing common pathological traits with *PPT1* (Haltia and Goebel, 2013).

## Materials and Methods

### Cell Culture

Human neuroblastoma SH-SY5Y cells (catalog number #94030304 European Collection of Cell Cultures) were cultured in DMEM-High glucose medium supplemented with 15% Foetal Bovine Serum (FBS), 2 mM L-glutamine and 1% non-essential amino acids (all from Euroclone), at 37°C in humified atmosphere with 5% CO_2_, as described (Pezzini et al., 2017).

### Production of PPT1 Constructs, Cell Transfection and Generation of Stable Cell Lines

We focused our attention on *CLN1* mutations described in Mediterranean patients affected by classical and variant CLN1 disease. Specifically, we selected three different missense mutations (c.665T>C/p.L222P, c.541G>A/p.V181M and c.541G>T/p.V181L), a deletion of the second exon (c.125_235del/p.G42_E78del) in which the ORF is maintained and a one-base insertion (c.169dupA/p.M57Nfs*45) predicting a frameshift, and a premature stop codon at residue 101 (Santorelli et al., 1998). Wild-type and mutated *CLN1* cDNAs (NM_000310) were inserted into pcDNA3 expression vector (Invitrogen, Life Technologies [LT]) by PCR methods. The sequences of *CLN1* constructs were confirmed by direct sequencing using BigDye Terminator v3.1 Cycle Sequencing Kit (Applied Biosystems, LT), on an ABI3130xl automatic DNA Analyzer. SH-SY5Y cells were plated 1 day before transfection at 80% confluence in 35 mm dishes, and then transfected with 250 ng of cDNAs per dish using lipofectamine method (Applied Biosystems, LT) following the manufacturer’s instructions. Clones which stably overexpressed the different *CLN1* cDNAs were finally isolated through antibiotic selection with 600 μg/ml geneticin (G418, Gibco, LT). Cells overexpressing the empty pcDNA3 vector (hereafter also referred as mock) served as a control (Table 1).

**Table 1:** The compendium of *CLN1* overexpressing SH-SY5Y neuroblastoma cell lines used in the study.

Cell line	Transfected *CLN1* cDNA	Effect on PPT1 protein
SH-SY5Y	-	-
SH-pcDNA3	pcDNA3 (Empty Vector)	-
SH-p.wtCLN1	wt*CLN1*	-
SH-p.L222P	c.665T>C	p.L222P
SH-p.V181L	c.541G>A	p.V181L
SH-p.V181M	c.541G>T	p.V181M
SH-p.M57Nfs*45	c.169dupA	p.M57Nfs*45
SH-p.G42_E78del	c.125+15T>G	p.G42_E78del

### Quantitative Real-Time RT-PCR Analysis

Total cellular RNA was extracted from neuroblastoma cells using the Tri Reagent (Sigma-Aldrich) following manufacturer’s instructions. The reverse transcription was performed by High-Capacity cDNA Reverse Transcription kit (Applied Biosystems, LT) and 1.5 μg of cDNAs were amplified in an ABI 7500 Fast real time PCR. The expression of *CLN1* was assessed by qPCR using inventoried Taqman assay (Hs00165579_m1; Applied Biosystems, LT), and normalized to the level of GAPDH (Hs99999905_m1) using the comparative Ct method.

### Neuronal Differentiation Paradigm

Parental SH-SY5Y cells and transfected cells were analyzed both under basal conditions and following neuronal differentiation in *all-trans* Retinoic Acid-Neurobasal medium (RA-NBM) medium (Pezzini et al., 2017). In brief, cells were pre-differentiated in DMEM/HIGH supplemented with 5% FBS and 10 μM RA (Sigma-Aldrich) for 6 days, and then grown for further 3 days in Neurobasal medium (GIBCO, LT) enriched with 50 ng/ml recombinant human BDNF (rhBDNF, Peprotech), 2 mM Dibutyryl-cyclic AMP (db-cAMP; Sigma-Aldrich), 20 mM KCl, B27 supplement and 1% GlutaMAX (GIBCO, LT). Control (not treated, NT) cells were cultured in parallel under basal conditions at the same FBS concentration (5%). Cells were seeded in T_75_ flasks or on coverslips coated with ECMax gel (Sigma-Aldrich) at the same density of 8 × 10^3^ cells per cm^2^, grown for 24–48 h and then exposed to RA-NBM treatment. Media were routinely changed every 2–3 days; the morphological features of control and *CLN1* transfected cell lines were monitored under an Axio Vert.A1 inverted microscope (Zeiss). The images were acquired by True Chrome HD II cam system. Cells grown under basal conditions were harvested after 6 days to avoid the culture over-growth.

### Immunofluorescence Microscopy

Following fixation in 4% paraformaldehyde, coverslips were rinsed in PBS and incubated in blocking solution and then probed overnight at 4°C with primary antibodies diluted in incubation buffer. Primary antibodies used for immunofluorescence (IF) assay were as follows: rabbit polyclonal anti-PPT1 (1:50, Cat# HPA021546, Sigma Aldrich), which recognized a C-terminal region (aa 223-306), mouse monoclonal anti-LAMP2 (1:400, H4B4 clone, Cat# ab25631, Abcam), mouse monoclonal SMI31R (specific for phosphorylated heavy chain neurofilament; 1:200, Cat# SMI31R, Covance), rabbit monoclonal anti-βIII tubulin (1:200, clone EP1569Y, Cat# 04-1049, Merck Millipore). Goat anti-mouse and anti-rabbit conjugated with AlexaFluor 488 or AlexaFluor 594 dyes (1:800, Cat# A11005 and A11008 respectively, Molecular Probes, LT) were used as secondary antibodies for 1 h at RT. Finally, nuclei were counterstained with 5 μg/ml DAPI (4′,6-diamidino-2-phenylindole dihydrochloride, Sigma-Aldrich). Images were acquired by an AxioLab microscope equipped with an AxioCam and AxioVision 4.3 software (Zeiss). In addition, confocal microscopy analyses following LAMP2/PPT1 staining was performed on a Zeiss LSM 710 (laser argon 488 nm and HeNe 543 nm) using 40× Plan NeoFluar 0.75 and 63× 1.4 NA Plan Apochromat oil-immersion lenses; images were acquired by Zeiss LSM 710 software and analyzed by ZEN Lite 2012 (Zeiss). Colocalization analysis of PPT1 and LAMP2 immunostainings was performed on SH-p.wtCLN1 cell line by Anima image analysis software (Rantanen et al., 2014).

The density of β-III tubulin immunolabeled neurites was evaluated by NeuronJ plugin of ImageJ as described elsewhere (Supplementary Methods in Supplementary Material and Pezzini et al., 2017). In brief, following images acquisition at 10× magnification, NeuronJ plugin was used to manually trace neurites; only processes longer than 30 μm were taken into account. The number of neurites was normalized on the cellular density by counting nuclei stained by DAPI. At least 1500 cells from three independent experiments were analyzed and data were reported as mean ± SEM; statistical significance was assessed by one-way ANOVA followed by Bonferroni post-test on SH-pcDNA3 cell line.

### Western Blotting Analysis

Cellular pellets were homogenized in RIPA buffer containing inhibitors of proteases (Roche Diagnostics GmbH), resolved by electrophoresis in SDS-polyacrylamide gels (or alternatively in NuPAGE Novex 4–12% Bis-Tris protein gels, LT) under denaturing conditions and finally transferred to PVDF membranes (Bio-Rad Laboratories). Primary antibodies used for Western Blotting (WB) are listed below: rabbit polyclonal anti-PPT1 (1:1000, Cat# HPA021546, Sigma Aldrich), mouse monoclonal anti-neurofilament 160/200 (1:500, Clone RMdO20, Cat# N2912, Sigma Aldrich), rabbit polyclonal anti-CRMP1 (1:2000, Cat# C2868, Sigma-Aldrich), rabbit polyclonal anti-GAP43 (1:2000, Cat# AB5220, Chemicon, Merck Millipore), rabbit polyclonal anti-SNAP25 (1:10,000, Cat# S9684, Sigma-Aldrich), rabbit polyclonal β-III tubulin (1:1000, Cat# CSB-PA03874A0Rb, Cusabio). Immunodetection with anti-GAPDH (1:30,000, Cat# G9545, Sigma-Aldrich) was used as loading control. Species-specific HRP conjugated antibodies (anti-mouse and anti-rabbit IgG HRP linked f(ab’)2 fragment, 1:10,000, Cat# GEHNA93101ML and GEHNA93401ML, Amersham GE Healthcare) were used as secondary antibodies. Chemiluminescent detection was performed with Immobilon Western Chemiluminescent HRP Substrate (Merck Millipore) according to manufacturer’s instructions. For quantitative evaluation, protein lysates from at least three independent experiments were assessed in the same electrophoretic run. For densitometry analysis, the optical density (OD) of each band was assessed by ImageJ software and normalized to OD of a loading control (GAPDH). Data were reported as mean ± SEM. Statistical analyses were carried out using either unpaired *t*-test analysis or ANOVA followed by Bonferroni *post hoc* test, considering as significant a *p* value < 0.05 and as highly significant a *p* value < 0.01.

### PPT1 Enzyme Activity Assay

PPT1 EA was assessed by monitoring fluorescence release of 4-methylumbelliferone in a LS-50B Perkin–Elmer fluorometer, as previously described (Bonsignore et al., 2006).

### Whole Transcriptomic Analysis by RNA-seq

The whole transcriptomic analysis was carried out on *CLN1* transfected SH-SY5Y cells following RA-NBM treatment. Specifically, we investigated the wt*CLN1* overexpressing cell line (SH-p.wtCLN1) and two cell lines carrying either a missense mutation (SH-p.L222P) or a mutation leading to premature protein truncation (SH-p.M57Nfs*45); empty-vector transfected cells (mock) and parental SH-SY5Y cells were also analyzed as a reference. RNA samples were collected in three independent experiments by TRI Reagent (LT) and checked for purity (by Nanodrop 1000, Agilent Technologies), and integrity (RNA Integrity Number ≥8.0). Indexed cDNA libraries’ preparation, sequencing by Illumina HiSeq 1000 sequencer and alignment of reads to reference human genome (hg19) were performed as previously reported (Pezzini et al., 2017). To allow the comparison of gene expression profiles, the normalized expression values for each transcript were calculated as Fragments Per Kilobase per Million mapped reads (FPKM). The expression profile of each *CLN1* transfected cell line (SH-p.wtCLN1, SH-p.L222P and SH-p.M57Nfs*45) was then compared to the profile of mock cells: for each gene the ratio between average FPKM of each cell line and average FPKM of mock cells was calculated and reported as log2 fold change (log2FC). Transcripts showing a |log2(FC)| ≥1 and a False Discovery Rate (FDR, *q*-value) ≤0.05 were assigned as differentially expressed.

### Bioinformatic Analysis and Categorization of Transcriptomic Data

Sets of differentially expressed genes (DEGs, corresponding to identified transcripts) of each *CLN1* transfected cell line, were evaluated by QIAGEN’s Ingenuity^®^ Pathway Analysis (IPA^®^, Winter release 2017, QIAGEN^2^), to recognize meaningful biological processes and molecular pathways. Specifically, we carried out a *Core Analysis* followed by *Downstream Effects Analysis*; Ingenuity Pathway Analysis (IPA) macro-categories of *Molecular and Cellular Functions* and *Physiological System Development and Function* were further scrutinized. *P*-value, which was ascertained by right-tailed Fisher’s Exact Test following Benjamini and Hochberg (B-H) correction, indicated the robustness of correlation between a subset of DEGs of the dataset with a given biological function. Moreover, IPA algorithm calculated a “*z*-score” which estimates a predicted activation or inhibition of a given biological function. In particular, to compare the three independent *Core Analyses* of SH-p.wtCLN1, SH-p.L222P and SH-p.M57Nfs*45, we selected the most meaningful functional annotations (*p*-value < 0.05) assigned to each *CLN1* transfected cell lines. Subsequently, only annotations demonstrating a |*z*-score| >0.5 in at least one cell line were taken into account for the heat-map representation. Furthermore, selected subsets of DEGs were also queried through PANTHER database (Protein ANalysis THrough Evolutionary Relationships, Mi et al., 2017)^3^ to elucidate the association with (GO) terms/attributes. Cytoscape software (version 3.4.0, Cline et al., 2007)^4^ with GeneMANIA Cytoscape plugin (Montojo et al., 2010) were utilized to draw networks.

### Identification of Differentially Expressed Genes Encoding for Palmitoylated Proteins

Datasets of DEGs from each *CLN1* expressing cell line were subsequently queried for genes encoding the palmitoylated proteins. Specifically, we inquired SwissPalm—Protein Palmitoylation database (Blanc et al., 2015)^5^ from which both evidence-based and predicted palmitoylated proteins were retrieved. In addition, we used the curated compendium of mammalian palmitoylome reported by Sanders et al. (2015), and containing 1838 candidate proteins identified by screening of 15 palmitoylation-related proteomic publications. Several DEGs encoding palmitoylated proteins, and assigned to relevant IPA biological functions were further investigated by semi-quantitative WB.

### Data Availability

RNA-seq data discussed in this publication have been deposited in NCBI’s Gene Expression Omnibus and are accessible through GEO Series accession number GSE98834^6^.

## Results

### Characterization of *CLN1* Transfected SH-SY5Y Cells

**Figure 1:**
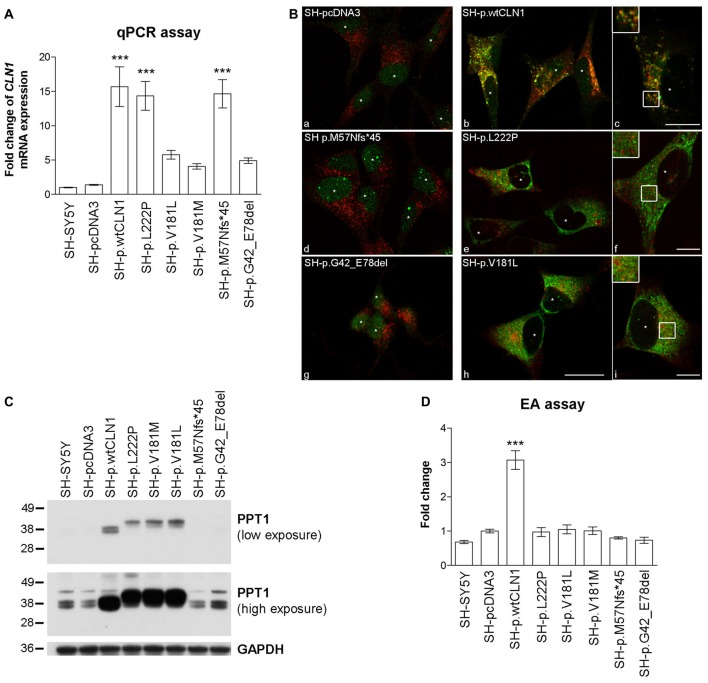
Characterization of *CLN1* mRNA and corresponding Palmitoyl-Protein Thioesterase 1 (PPT1) protein expression in *CLN1* transfected cells. **(A)** qRT-PCR quantitation of mRNA expression in transfected *CLN1* cells. All transfected cells showed an increased expression of *CLN1* mRNA ranging from 5 to 15 fold change in comparison to the endogenous level in controls (both parental and empty vector pcDNA3, mock-transfected cells). Mean ± SEM of three independent experiments; One-way ANOVA followed by Bonferroni’s multiple comparison test; ***p < 0.001. **(B)** Double immunofluorescence (IF) assay for PPT1 (green) and the lysosomal marker LAMP2 (red) revealed a strong signal of PPT1 in cells which stably expressed either wt*CLN1* (b and c) or two different missense mutations (p.L222P, e and f; p.V181L, h and i). Empty vector expressing cells (a), the *CLN1* insertion (p.M57Nfs*45, d) and the *CLN1* deletion (p.G42_E78del, g) are shown in comparison. A partial localization inside lysosomes was observed for SH-p.wtCLN1 (inset in c), whereas a more diffuse cytoplasmic pattern was detected for SH-p.L222P and SH-p.V181L (f and i). Nuclei are marked by white asterisks; scale bars equal to 20 μm (h) and 10 μm for insets (c, f and i). Colocalization is shown in yellow. **(C)** Overexpression of PPT1 in both SH-p.wtCLN1 cells and cell lines harboring a missense mutation was confirmed by immunoblotting analysis. The 38–36 kDa doublets were detected in homogenates of wt*CLN1* cells. A different pattern was associated with the missense mutation-bearing proteoforms of PPT1- a single band, running approximately at 41 kDa, was detected in homogenates of these *CLN1* mutants only. In cells transfected with *indel* mutations, the 38–36 kDa doublets could be identified following high exposure only, suggestive for a representation of the endogenous PPT1 signal. NT, non-treated; *all-trans* Retinoic Acid-Neurobasal medium (RA-NBM), differentiated cells. **(D)** Strong increase in PPT1 enzymatic activity (EA) was observed in SH-p.wtCLN1 as compared to SH-pcDNA3 empty vector cells (3-fold change). Parental SH-SY5Y and cell lines carrying different mutations demonstrated some variability in PPT1 EA, with less than 0.25 fold change in relation to the empty vector transfected cell line. Mean ± SEM of three independent experiments; One-way ANOVA followed by Bonferroni’s multiple comparison test; ***p < 0.001.

To assess the overall efficacy of *CLN1* transfection, we investigated the expression of *CLN1* by quantitative real-time PCR (qPCR) performed on RNA samples derived from different neuroblastoma clones. *CLN1* mRNA overexpression ranged from 5 to 15- fold change as compared to basic expression in parental SH-SY5Y line and empty vector (pcDNA3) transfected cells. *CLN1* mRNA overexpression was significantly high in three generated cell lines, namely SH-p.wtCLN1, SH-p.L222P, SH-p.M57Nfs*45 (Figure 1A).

The expression of PPT1 was subsequently characterized by IF, WB and EA. Following IF, both parental and mock-transfected SH-SY5Y cells demonstrated a faint cytoplasmic dot-like staining (Figure 1B). A clear dot-like pattern was associated with the overexpression of wtPPT1, which partially colocalized with LAMP2 lysosomal staining (Overlap coefficient equal to 0.59, in accordance with other wtPPT1 cellular models; Scifo et al., 2015a,b); in addition, wtPPT1 was partially detected inside LAMP2 positive vesicles (inset c in Figure 1B). Conversely, a more diffuse, cytoplasmic pattern associated with a reduced PPT1 localization in LAMP2-immunolabeled vesicles was observed in cells carrying missense mutations. Colocalization analysis confirmed the qualitative findings and showed lower Overlap coefficients of missense mutated PPT1 with the lysosomal staining as compared to the wtPPT1 (Supplementary Figure S1). Cells transfected with cDNA harboring either the deletion (SH-p.G42_E78del) or the single base insertion (SH-p.M57Nfs*45) showed a faint IF, as detected in parental and mock-transfected cells (Figure 1B).

WB revealed differences in the patterns of expression of PPT1. In the lysates of parental and mock-transfected cells, anti-human PPT1 antibody recognized three bands running approximately at 43, 38 and 36 kDa (Figure 1C). The 38–36 kDa doublet likely represented mono- and di-glycosylated isoforms of mature PPT1, as previously described in neuronal cells (Lyly et al., 2007; Scifo et al., 2015a), whereas the ~43 kDa band—present to a similar extent in all the analyzed cell lysates—may denote an uncharacterized form of PPT1 specific for SH-SY5Y cells (Scifo et al., 2015a). SH-p.wtCLN1 demonstrated a remarkable increase in the expression of the 38–36 kDa doublet whereas the 43 kDa band remained unchanged. Upon treatment with PNGase, performed to remove glycosylated chains, an apparent shift of about 4 kDa in mass resulted in a single PPT1-immunoreactive band in SH-p.wtCLN1 (Supplementary Figure S2).

Cell lines harboring missense mutations displayed highly intense signal of PPT1 protein, but the electrophoretic mobility pattern was clearly different in comparison to wt*CLN1*; a new band migrating approximately at 41 kDa was the most intense whereas the 38–36 kDa doublet was weaker. Deglycosylation following PNGase treatment resulted in a single band, shifted approximately by 1–2 kDa, in p.L222P and p.V181L (Supplementary Figure S2). The weak 38–36 kDa doublets, consistent with the expression of endogenous PPT1, were detectable in the *indel* clones (SH-p.G42_E78del and SH-p.M57Nfs*45).

Furthermore, we checked whether the overexpressed PPT1 forms retained the enzymatic activity. Mock cells showed a slight increase (~40%) in PPT1 activity as compared to parental SH-SY5Y cells. On the contrary, cells overexpressing wt*CLN1* exhibited a 3-fold higher enzymatic activity, in relation to mock-transfected SH-SY5Y cells. Activities measured in other *CLN1* cell lines showed values ranging between parental and SH-pcDNA3 values, suggesting that only the endogenous activity of PPT1 was retained in these cells (Figure 1D).

The neuronal differentiation paradigm in RA-NBM media was effective in all analyzed cell lines, as reported for the parental SH-SY5Y neuroblastoma cells (Pezzini et al., 2017). Changes in shape of neuronal cell bodies and a significant elongation of their main processes have occurred (Supplementary Figure S3A), followed by alterations in the expression of SEM3A, a protein related to the induction of neuronal differentiation (conversion to a lower 65 kDa isoform, Supplementary Figure S3B). The expression of PPT1 was not affected by differentiation in RA-NBM media (Supplementary Figure S3C).

### Comparative Analysis of Gene Expression Profiles

**Figure 2:**
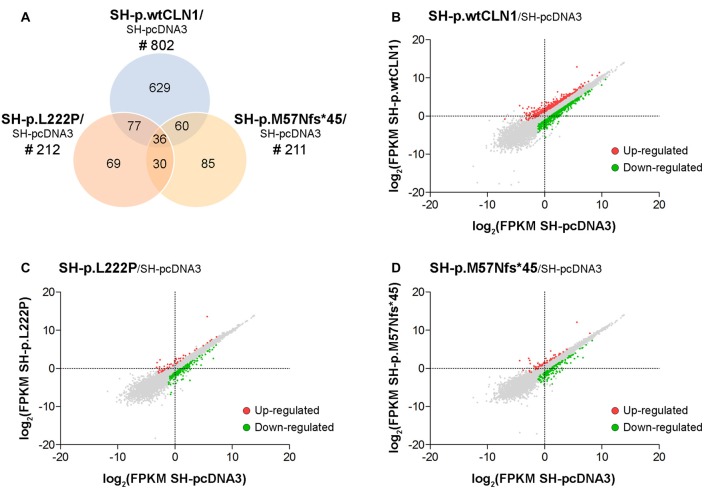
Transcriptomic analysis of *CLN1* transfected cell lines. **(A)** Venn diagram demonstrating the amount of differentially expressed genes, identified in SH-p.wtCLN1 cells (*n* = 802) and in the two mutated *CLN1*-transfected cell lines (*n* = 212, SH-p.L222P and *n* = 211, SH-p.M57Nfs*45). Notably, 629 differentially expressed genes (DEGs) were exclusively expressed in the SH-p.wtCLN1 clone. Differential gene expression analysis was performed on RA-NBM differentiated cell lines and compared to the empty vector expressing cells (SH-pcDNA3), analyzed under the same culture conditions. **(B–D)** Scatter plot representation of differentially expressed genes identified in differentiated SH-p.wtCLN1 **(B)**, SH-p.L222P **(C)** and SH-p.M57Nfs*45 **(D)** cells. Gene expression levels are reported as log2(FPKM) (*y* axis) and compared to differentiated SH-pcDNA3 cells (*x* axis). Colored dots represent differentially expressed genes (thresholds: |log2FC| > 1 and *q*-value < 0.05), which are either up-regulated (red dots) or down-regulated (green dots).

We generated a transcriptomic profile of three *CLN1* transfected cells as compared to empty-vector pcDNA3 expressing cells. We chose three cell lines (SH-p.wtCLN1, SH-p.L222P, SH-p.M57Nfs*45), which expressed the highest amount of *CLN1* mRNA upon transfection (Figure 1A). The transcriptomic profiles revealed 802 transcripts differentially expressed in SH-p.wtCLN1 cells (of which 286 were up-regulated and 516 down-regulated, Supplementary Table S1). The number of differentially expressed transcripts identified in the two mutated clones was significantly lower (212 for SH-p.L222P, 50 up- and 162 down-regulated, Supplementary Table S2; 211 for SH-p.M57Nfs*45, 54 up- and 157 down-regulated, Supplementary Table S3). 629 transcripts were specifically dysregulated in cells overexpressing the wild-type form of PPT1 (Figure 2).

### Bioinformatic Examination of Differentially Expressed Genes

**Figure 3:**
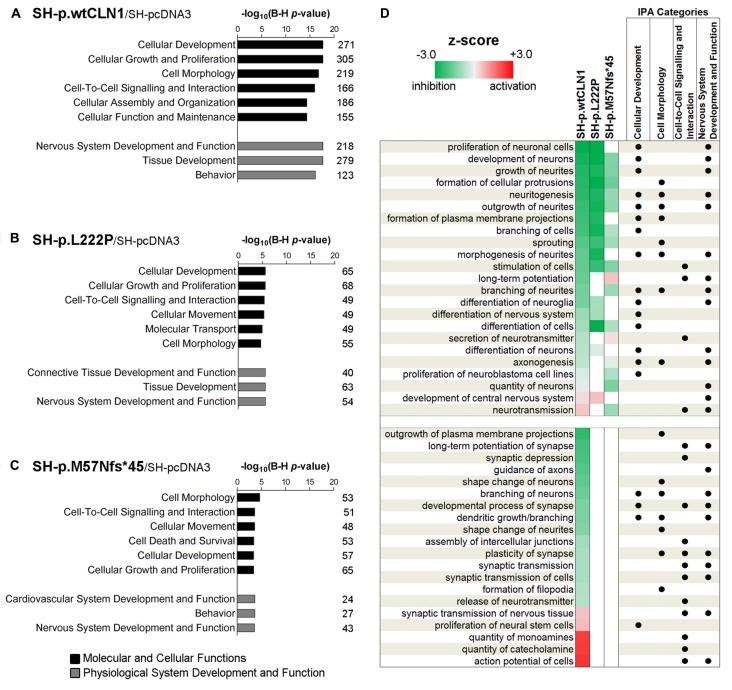
Bioinformatic survey of identified differentially expressed genes in wild-type and mutated *CLN1* cell lines. **(A–C)** Categorization by Ingenuity Pathway Analysis (IPA, Qiagen) of DEGs in differentiated SH-SY5Y cells. *Molecular and Cellular Functions* as well as *Physiological System Development and Functions* IPA classes were examined. Categories of *Cellular Development*, *Cell-to-Cell Signalling and Interaction*, *Cell Morphology* as well as *Nervous System Development and Function* with lowest B-H corrected *p*-values were further scrutinized. The number of genes assigned to each category is reported. Statistical significance is reported as –log_10_ of Benjamini-Hochberg corrected *p*-value. **(D)** Heat-map representation depicted most meaningful IPA functional annotations enclosed in *Cellular Development*, *Cell-to-Cell Signalling and Interaction*, *Cell Morphology* and *Nervous System Development and Function*. Cellular functions correlating with morphological changes of neuronal cells (*neuritogenesis, growth/branching/ morphogenesis of neurites* and *axonogenesis*) and neuronal commitment (*differentiation of neurons, development of neurons*) were commonly affected in SH-p.wtCLN1 and, to a lesser extent, in the two mutated cell lines, following a gradient according to *z*-scores (from highest to lowest). Functions related to neuronal transmission (*long-term potentiation, secretion of neurotransmitter and neurotransmission*) were also annotated. Notably, other annotations related to sprouting of neuronal processes (*dendritic growth/branching, shape change of neurites*, *shape change/branching of neurons*, *guidance of axons*) as well as synaptic transmission (*developmental process of synapse, long-term potentiation of synapse, plasticity of synapse, release of neurotransmitter* and *action potential of cells*) were selectively assigned to SH-p.wtCLN1 cells only. Functional annotations, shared among the IPA classes are marked by dots. Colored squares represent either a predicted activation (red) or a predicted inhibition (green) according to *z*-score calculated by IPA algorithm; higher color intensity correlates with more significant bioinformatic prediction.

Categorization through the bioinformatic suite IPA of DEGs identified in SH-p.wtCLN1 demonstrated *Cellular Development*, *Cell-to-Cell Signalling and Interaction* and *Cell Morphology* as the most meaningful *Molecular and Cellular Functions* categories. Moreover, *Nervous System Development and Function* was among the three high-ranked categories related to the *Physiological System Development and Function*, suggesting an alteration of neurobiological processes in these cells (Figure 3A). Likewise, the same IPA categories were significantly annotated in the two mutants (Figures 3B,C). SH-p.wtCLN1 cells displayed a higher number of DEGs which were assigned to each IPA category. Further scrutiny of the four main categories in SH-p.wtCLN1 profile pinpointed functional annotations related to changes of neuronal cell shape and formation of neurites (*neuritogenesis; growth, branching and morphogenesis of neurites; axonogenesis*) as well as neuronal differentiation (*differentiation of neurons, development of neurons*; Figure 3D). Taking into account the *z*-scores, most of these annotations were severely affected in SH-p.wtCLN1 and predicted to be inhibited; a similar trend was seen for SH-p.L222P (even though the number of annotated functions was lower than wt*CLN1* clone), whereas bioinformatic predictions for SH-p.M57Nfs*45 were less pronounced. Functions related to neuronal transmission (such as *long-term potentiation, secretion of neurotransmitter* and *neurotransmission*) were also annotated. Additional IPA attributes referred to sprouting of neuronal processes (*dendritic growth/branching, shape change of neurites*, *shape change/branching of neurons*, *guidance of axons*) as well as synaptic transmission (*developmental process of synapse*, *long-term potentiation of synapse, plasticity of synapse, release of neurotransmitter* and *action potential of cells*) were selectively assigned to SH-p.wtCLN1 cells (lower part of the Figure 3D). Notably, both the relative high number of annotations and associated *z*-scores indicated that these functions were remarkably affected in SH-p.wtCLN1 as compared to other analyzed cell lines.

### Palmitoylation Gene/Protein Survey

**Figure 4:**
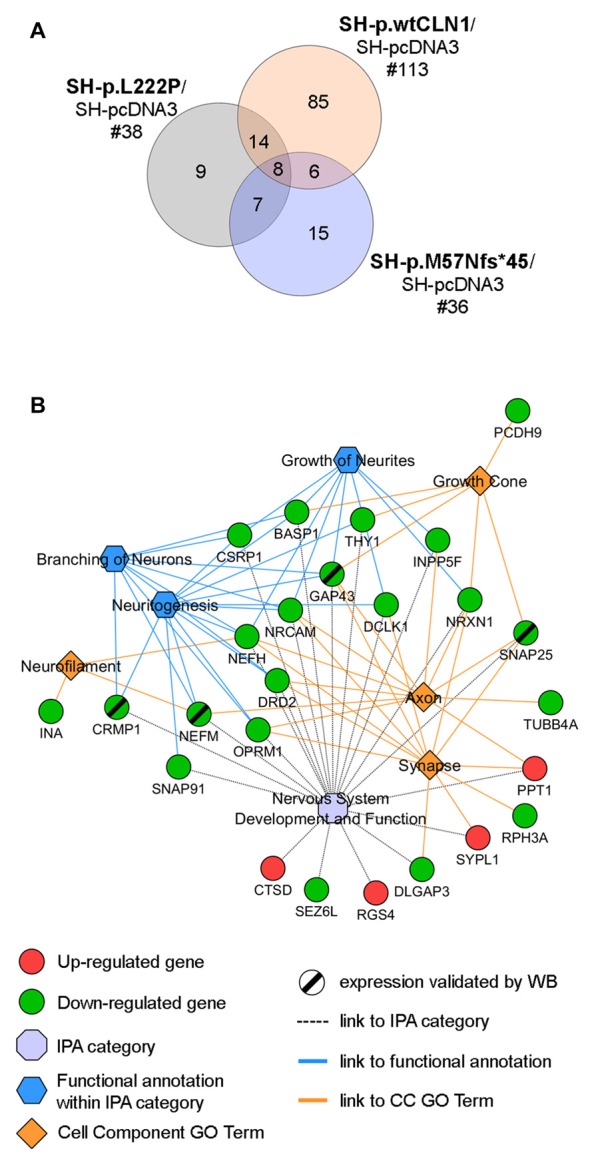
Palmitoylation gene/protein survey. **(A)** Venn diagram of identified palmitoylated gene products (palmitoylated proteins) among differentially expressed genes in the transcriptomic profiles of SH-p.wtCLN1, SH-p.L222P and SH-p.M57Nfs*45, compared to an empty-vector (SH-pcDNA3) expressing cells. The amount of identified DEGs is stated below the cell line name, whereas the numbers of genes shared among the three comparisons are reported in the intersections of the Venn diagram. Eighty-five of such genes were selectively expressed in SH-p.wtCLN1 only. See Supplementary Tables S4, S5, S6 for the compendium of genes of each *CLN1* cell line and Supplementary Figure S4 for genes assigned to each interaction area of Venn diagram. **(B)** Twenty five genes, specifically expressed in SH-p.wtCLN1 cells, were connected to *Nervous System Development and Function* as well as to annotations related to neurites formations. Cell Component GO terms (PANTHER) were also linked. Notably, *CTSD*, associated with CLN10 disease was found to be up-regulated in wild-type *PPT1*-overexpressing cells, further supporting a genetic interaction between these two neuronal ceroid lipofuscinoses (NCL) genes. For an exhaustive compendium of identified genes encoding the palmitoylated proteins and assigned IPA functions and GO terms see Table S7 in supplementary materials. Genes, marked by a black slash were further investigated for protein expression by Western Blotting (WB) analysis.

Taking into account the process of palmitoylation in which *CLN1/PPT1* is involved, we further scrutinized DEGs identified in the three *CLN1* transfected cell lines to seek for genes which are known to encode palmitoylated proteins. Survey of the mammalian palmitoylome derived from Blanc et al. (2015) and Sanders et al. (2015) in SH-p.wtCLN1 revealed 113 palmitoylated protein-encoding genes, corresponding to ~14% of the full DEGs dataset in this cell line (Supplementary Table S4). Conversely, a lower amount of DEGs coding for palmitoylated proteins was identified in the two mutants (*n* = 38 for SH-p.L222P, 18% of DEGs; *n* = 36 in SH-p.M57Nfs*45, 17% of DEGs; Supplementary Tables S5, S6). Eighty-five genes coding for palmitoylated proteins were specifically found in p.wtCLN1, whereas only a small portion was selectively expressed in the two mutants, (*n* = 9 in p.L222P and *n* = 15 in p.M57Nfs*45). Eight genes were common to all mutated cell lines (see Venn diagram in Figure 4A and network analysis in Supplementary Figure S4).

Bioinformatic categorization of 113 palmitoylated protein-encoding genes seen in SH-p.wtCLN1 using PANTHER revealed a significant enrichment in Cell Component GO terms related to *Neuron projection, Neurofilament, Growth cone, Axon* as well as *Synapse* (Supplementary Figure S5). Furthermore, 76 genes were assigned to the main four, previously described IPA categories; likewise, 25 genes were assigned to functional annotations related to formation and elongation of neuronal processes, such as *neuritogenesis*, *branching of neurons* and *growth of neurites* (Table S7 in supplementary Materials). Confined collection of these 25 genes, shared among relevant IPA functional annotations and Cell Component GO terms, is outlined in Figure 4B.

### Gene-Product Expression of Selected DEGs

**Figure 5:**
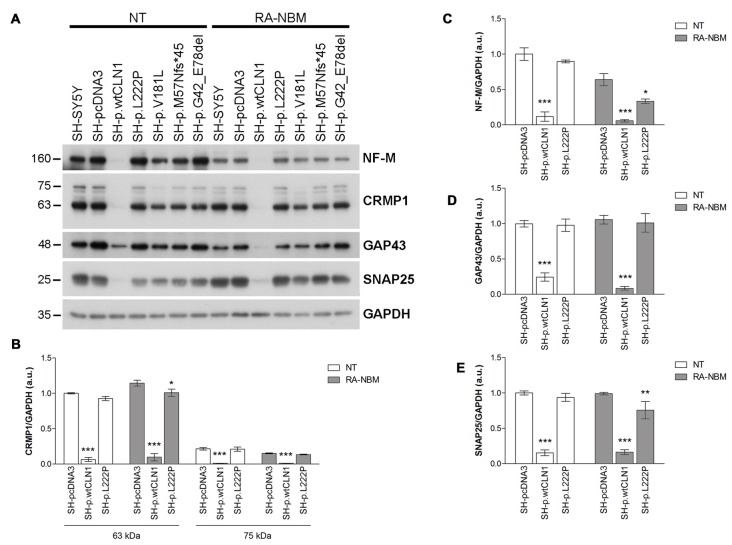
Immunoblotting analysis of selected DEGs identified in SH p.wtCLN1 cells and encoding for palmitoylated proteins. **(A)** Representative WB of proteins coded by four down-regulated genes (NF-M, CRMP1, GAP43 and SNAP25), identified in SH p.wtCLN1 transcriptomic profile. A strong decrease in protein expression was evident in SH-p.wtCLN1 cellular lysates, in comparison to no visible effect in other cell lines. **(B–E)** Semi-quantitative WB analysis of the same proteins confirmed highly decreased expression of these markers in SH-p.wtCLN1 cells, both under basal condition and following differentiation in RA-NBM medium. A slight down-regulation in expression of a short 60 kDa isoform of CRMP1 (isoform 2), NF-M and SNAP25 following RA-NBM differentiation was detected in SH-p.L222p lysates whereas no significant change in GAP43 expression was observed in the same cell line. GAPDH served as internal standard; a.u. arbitrary units; mean ± SEM of three independent experiments; Two-way ANOVA followed by Bonferroni’s post-test; *p < 0.05, **p < 0.01, ***p < 0.001.

Among DEGs coding for palmitoylated proteins detected in SH-p.wtCLN1 cells, we selected genes relevant to elongation of neuronal structures (*GAP43*, *CRMP1* and *NEFM*) and assessed their protein expression by WB. Immunoblotting showed a decreased expression of the corresponding proteins in cellular lysates of SH-p.wtCLN1, whereas no significant changes in the other mutated *CLN1* cell lines were observed (Figure 5A). A similar trend was seen for the synaptic marker *SNAP25*, another DEG specifically identified in SH-p.wtCLN1. The quantification analysis in SH-p.wtCLN1 and other cell lines (Figures 5B–E) confirmed a significant down-regulation of these markers in SH-p.wtCLN1, and to a lesser extent in SH-p.L222P cells. A reduced expression of these proteins was already evident under basal conditions, suggesting that down-regulation of these proteins (and transcripts) is not related to the RA-NBM differentiation process.

### Impaired Neuritogenesis: Morphological Features of *CLN1* Transfected SH-SY5Y Cells

**Figure 6:**
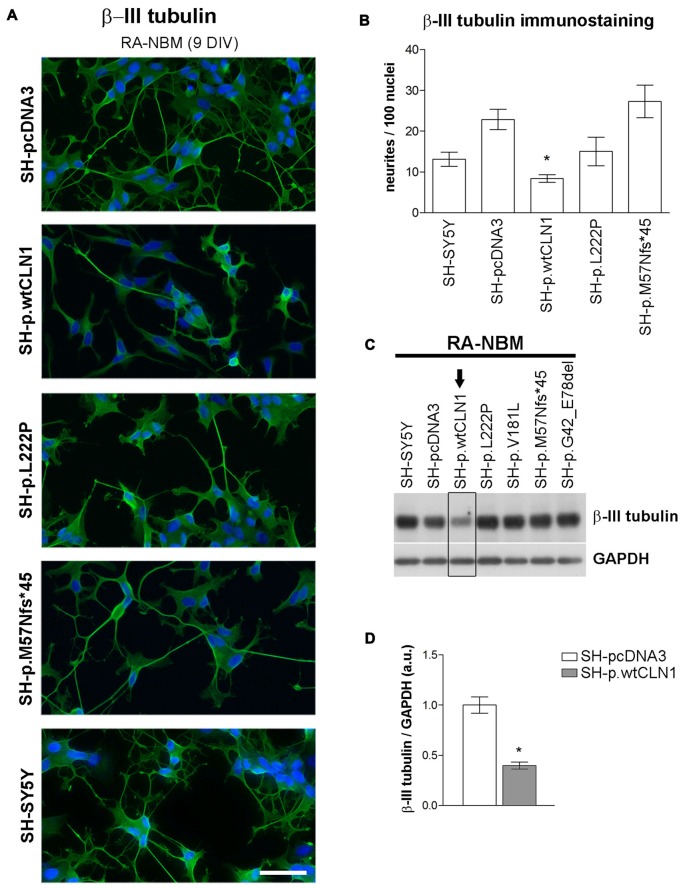
Neurite growth features of *CLN1* transfected cell lines following neuronal differentiation in RA-NBM medium. **(A)** Outgrowth of neurites in *CLN1* transfected cells lines were evaluated by β-III tubulin immunostaining during neuronal differentiation. SH-p.wtCLN1 cells showed less arborized, stunted neurites in comparison to mock-transfected cells and other *CLN1* transfected cell lines. Nuclei are marked in blue; DIV, days *in vitro*; scale bars equal to 50 μm. For a qualitative evaluation of cell morphology see also Figure S6 in the supplementary Materials. **(B)** The morphometric analysis of β-III tubulin immunolabeled processes pinpointed a significant paucity of neurites longer than 30 μm in SH-p.wtCLN1 cells, in accordance with the bioinformatic findings. Likewise, a similar, even though not significant, trend was seen for SH-p.L222P clone. Higher variability was detected in SH-p.M57Nfs*45. These findings resembled the results about neurofilament immunolabeled structures which are described in Figure S7 of supplementary Materials. Mean ± SEM of three independent experiments; One-way ANOVA followed by Bonferroni’s post-test; *p < 0.05. **(C)** Immunoblotting analysis of β-III tubulin demonstrated a reduced expression of this cytoskeletal marker in SH-p.wtCLN1 only, in accordance with the decreased number of neurites. Arrow pinpoints to a decreased expression of β-III tubulin in SH-p.wtCN1 cells. **(D)** Semi-quantitative WB analysis confirmed a decreased expression of β-III tubulin in SH-p.wtCLN1, following differentiation in RA-NBM medium. GAPDH served as internal standard; a.u. arbitrary units; mean ± SEM of three independent experiments; unpaired *t*-test; *p < 0.05.

Transcriptomic analysis and bioinformatic investigations suggested a defective capacity of SH-p.wtCLN1 cells to generate elongated cellular processes. We thus evaluated the morphological changes undergoing in *CLN1* transfected cells during the differentiation process in RA-NBM medium. Under basal conditions, all analyzed cells showed similar morphological features and growth characteristics, except those overexpressing wt*CLN1*, which exhibited a slow rate of proliferation and a more flattened shape. Differentiation in RA-NBM medium significantly stimulated the outgrowth of neuronal processes in all cell lines (Supplementary Figure S6). However, SH-p.wtCLN1 revealed a peculiar behavior: cells shrank their cytoplasm, became more polarized and extended some membrane processes during exposure to RA medium for the first 6 days. At the same time, these cells seemed to demonstrate a less triangular/branched shape in comparison to the other cell lines. The exposure to NBM medium enriched with neurotrophic factors for further 3 days increased the complexity and arborization of the elongated structures in the different mutant cell lines and controls, whereas wt*CLN1* cells exhibited processes which appeared more stunted and less arborized (Supplementary Figure S6). Considering the predicted defect of *neuritogenesis*, we subsequently tested the expression of β-III tubulin, a cytoskeletal marker of neurites. Most of the arborized neurites were heavily immunostained by β-III tubulin antibody in differentiated SH-SY5Y, SH-pcDNA3 and SH-p.M57Nfs*45 cells whereas fewer processes were detected in SH-p.wtCLN1 following quantitative morphometric evaluation (Figures 6A,B). Accordingly, we observed a reduced expression of β-III tubulin in differentiated SH-p.wtCLN1 (Figures 6C,D). Likewise, the immunofluorescent analysis of pNF-H immunolabeled structures indicated that SH-p.wtCLN1 cells (and to a lesser extent SH-p.L222P cells) were characterized by less frequent axonal-like processes, as compared to mutant and mock-transfected cells (see Supplementary Material and Methods and Supplementary Figure S7).

### Synaptic Compartment-Related Genes in SH-p.wtCLN1 Cells

**Figure 7:**
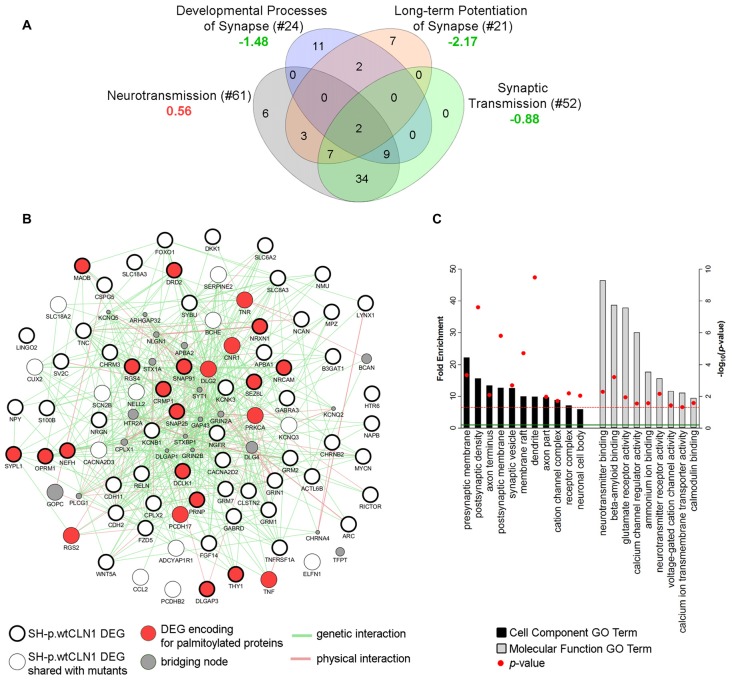
Bioinformatic survey of DEGs assigned to the synaptic compartment. **(A)** Four IPA functional annotations of SH-p.wtCLN1 transcriptomic profile, and related to neuronal transmission were investigated. The number of DEGs assigned to each category is reported between brackets whereas the associated *z*-score is reported below (green = predicted inhibition; red = predicted activation). The four IPA attributes enclosed 81 unique DEGs, which served as input for further bioinformatic queries. **(B)** A functional network encompassing 81 DEGs drawn using GeneMANIA plugin in Cytoscape software. Both genetic and physical interactions were used to denote the bridging nodes. Thick borders pinpoint DEGs which were specifically expressed in SH-p.wtCLN1, and not shared with the other two mutants. Genes encoding for palmitoylated proteins are marked in red. *GAP43* and *BCAN* nodes represent down-regulated genes of SH-p.wtCLN1 transcriptomic profile. *GAP43* encodes for a membrane protein associated with a growth cone of neuronal cells (see discussion section for further information), whereas *BCAN* encodes brevican, a chondroitin sulfate proteoglycan highly expressed in the extracellular matrix of synaptic space (Blosa et al., 2015). **(C)** Statistical overrepresentation profile of 81 DEGs derived through PANTHER classification system linked them to synaptic compartments as well as axons (Cell Component GO terms categorization). Likewise, many genes were involved in membrane channel activity and regulation, as well as in neurotransmission and glutamate receptor activity (Molecular Function GO terms). Fold Enrichment (FE) score represents a portion of genes enclosed in a specific term. Enrichment above a score of 1 (marked by green line) indicates an overrepresentation. GO terms were grouped for related ontological classes and sorted hierarchically according to a decreasing FE score, starting from the most specific subclass towards less specific ones. Only the most specific categories for each class associated with a FE score greater than the expected value are shown. *P*-value was adjusted using Bonferroni correction for multiple testing and reported as −log_10_(*p*-value); red line represents a *p*-value equal to 0.05.

Transcriptomic profiling and bioinformatic inquiry of DEGs identified in SH-p.wtCLN1 put forward several functional annotations related to synaptic compartment and synapse function (Figure 3D). Therefore, we focused on four IPA annotations related to neurotransmission (81 DEGs), namely *developmental process of synapse* (*z*-score = −1.48), *long-term potentiation of synapse* (*z*-score = −2.17)*, synaptic transmission* (*z*-score = −0.88) and *neurotransmission* (*z*-score = 0.56; see Venn diagram in Figure 7A). Further inquiry through GeneMANIA revealed close associations between DEGs as far as *genetic* and *physical interactions* were concerned (Figure 7B). Moreover, fold enrichment analysis through PANTHER disclosed important functional relations of those DEGs with synaptic and axonal compartments (Cell Component GO terms of *pre/post-synaptic membrane*, *axon terminus* and *synaptic vesicle*, Figure 7C) as well as with regulatory activities of membrane channels (Molecular Functions GO terms, Figure 7C). Specifically, various genes were assigned to *glutamate receptor activity* (*GRM1*, *GRM2*, *GRM7*, *GRIN1*), *calcium channel regulator activity* (*GRM2*, *NPY*, *GRM7*, *NRXN1*) and *voltage-gated cation channel activity* (*KCNB1*, *OPRM1*, *KCNQ3*, *CACNA2D2*, *KCNK3*, *GRM7*). Among queried DEGs, 23 genes encoded palmitoylated proteins (red nodes in Figure 7B and Supplementary Table S4).

## Discussion

In this experimental setting, an increased expression of a wild-type *CLN1* was functionally linked to DEGs encoding palmitoylated neuronal proteins, pinpointed impaired cellular functions (i.e., neuritogenesis) and coupled with dysregulated expression of synapse-related genes. Affected modules and related functions are associated with the pathological effects related to loss-of-function, and are known to be affected in *Ppt1*^−/−^ mice, including pathological changes of a synaptic compartment (Virmani et al., 2005; Kielar et al., 2009; Peng et al., 2015; Tikka et al., 2016); moreover a severe epilepsy associated with typical Electroencephalogram (EEG) pattern (Santavuori, 1973) occurs in CLN1 patients, who are lacking PPT1 functional activity.

### The Effects of Transfection on PPT1 Expression and Cellular Differentiation

Characterization of the *CLN1* transfected clones revealed three patterns of expression (Table 1 and Figure 1). Despite the successful transfection of *CLN1* constructs, no enhanced PPT1 signal was observed on immunoblots of SH-SY5Y cells harboring the p.M57Nfs*45 and p.G42_E78del mutations. It is tempting to speculate that this finding was related to the ablation of about two thirds (p.M57Nfs*45), or excision of 36 residues (p.G42_E78del) of a PPT1 protein, and a likely degradation before post-translational processing is being completed (Bravo et al., 2013). Alternatively, the deletion may affect the conformation/folding properties of PPT1. In accordance with both hypotheses, we could not measure the PPT1 enzymatic activity above the endogenous level. Cells harboring a missense mutation (p.L222P, p.V181L or p.V181M) overexpressed a unique band migrating approximately at ~41 kDa; a different electrophoretic pattern was also confirmed upon de-glycosylation with PNGase as compared to the de-glycosylated wtPPT1 isoform. The shift in the apparent molecular weight may indicate a full-length PPT1, retaining the N-term signal peptide (27 amino acids), which in turn may also be hyper-glycosylated. It is not clear how these changes occur, nor how they affect the catalytic domains of PPT1, as suggested by lack of enzymatic activity observed for the overexpressed, missense mutations bearing PPT1 proteoforms (Bellizzi et al., 2000). Lastly, in SH-p.wtCLN1 overexpressing cells the two glycosylated isoforms of mature PPT1 (38–36 kDa doublet, mono- and di-glycosylated forms, respectively) could be detected (Lyly et al., 2007; Scifo et al., 2015a). In accordance with the pattern of expression on WB, a three-fold increase in EA was observed (Figure 1D).

Normal processing of an overexpressed PPT1 in the SH-p.wtCLN1 cells was suggested by IF where the identification of PPT1 signal along axonal-like structures and in peripheral regions (synaptic terminal and varicosities) of differentiated cells was in accordance with outbound delivery of the protein, similarly as described in other neuronal cell lines (Ahtiainen et al., 2003). These patterns were not seen in missense clones consistent with defective processing and mislocalization of the mutated PPT1. Lastly, the used experimental conditions did not affect the rate of cell death in any clone tested (unpublished observations), in accordance with findings previously described in LAN-5 neuroblastoma cell line (Cho and Dawson, 2000).

### RNA-seq Analyses

Our experimental findings indicated that the number of DEGs was related to the amount of mutated *CLN1* mRNA, and to its’ ability to be translated into a functional protein. These findings were supported by the number of identified DEGs, which was similar among the two analyzed, mutated cell lines (~210 DEGs), but far less in comparison to the overexpressing wtCLN1 clone (>800 DEGs). Bioinformatic analysis of differentially expressed transcripts revealed functional links related to changes of neuronal cell shape and formation of neurites, which were predicted to be impaired in SH-p.wtCLN1; a similar trend (with less annotated functions) was seen for SH-p.L222P. Accordingly, the morphometric analysis pinpointed a reduced capacity of the same cell lines, to generate primary axonal processes. Nonetheless, the bioinformatic profiles of the two mutated clones were different: the expression of the mutated protein (SH-p.L222P clone) affected the expression of few genes which impacted on the cell morphology, in partial overlap with SH-p.wtCLN1, whereas a markedly reduced number of annotations was associated with the SH-p.M57Nfs*45 clone, inept to yield any functional PPT1 proteoform.

Functional annotations related to defective outgrowth of neuronal structures (including neuritogenesis and sprouting) as well as axonal degeneration have also been described in *Ppt1*^−/−^ mice at the pre-symptomatic stage (Tikka et al., 2016), and during the early phase of embryonic neuronal development in a fly model of CLN1 disease (Chu-Lagraff et al., 2010).

Moreover, a second meaningful functional module of SH-p.wtCLN1 profile was related to the synaptic compartment and synapse physiology annotations (Figures 3D, 7), and may be consistent with the extra-lysosomal role of PPT1 in the nerve terminals (Lehtovirta et al., 2001; Kim et al., 2008). A prospective role of PPT1 in modulating the function of membrane proteins related with neuronal excitability and, possibly, epileptogenesis requires evidence gained by other experimental approaches.

### Palmitoylation Gene Survey

We recognized 113 genes in SH-p.wtCLN1 dataset that encoded palmitoylated proteins, of which 76 genes were further assigned to relevant molecular and cellular functions (Supplementary Table S4). Our results indicate that hyper-depalmitoylating activity may perturb the expression of proteins relevant to neuritogenesis and synaptic transmission. The precise relationship underlying the apparent negative feedback between an overexpressed depalmitoylating protein and genes encoding palmitoylated proteins is not yet known. However, it has been recently demonstrated that PPT1 itself is regulated by the palmitoylation at cysteine residue in position 6 of the signal peptide, creating a sort of a positive feedback loop (Segal-Salto et al., 2016).

Twenty-five DEGs were of greater interest because they encode palmitoylated proteins, assigned to both neuronal branching and synaptic physiology. The downregulation in wt*CLN1* overexpressing cells of four proteins, namely neuromodulin (GAP43), CRMP1, Neurofilament-M (NF-M) and SNAP25, was corroborated by WB in both basal conditions and following RA-NBM treatment. The role of GAP43, CRMP1 and NFs in the elongation of cellular projections and pathfinding processes have been extensively characterized in neuronal cells. GAP43 localizes to the growth cones and synaptic terminals of elongating axons and is mainly involved in the modulation and arborization of growing neuronal structures (Meiri et al., 1986; Benowitz and Routtenberg, 1997). Both lipidation through palmitoylation and phosphorylation regulate the localization and diffusion of GAP43 on the plasma membrane, in the terminal tips of growing axons (Liu et al., 1994; Strittmatter et al., 1994; Trenchi et al., 2009; Gauthier-Kemper et al., 2014).

CRMP1, a member of the the Collapsin Response Mediator Proteins (CRMPs) family, regulates many aspects of cytoskeletal remodeling and arborization of neurites and dendrites in neuronal cells (Yamashita and Goshima, 2012; Quach et al., 2015; Nagai et al., 2017). Phosphorylation status of CRMP1 affects the signaling pathways mediated by Semaphorin 3A (SEMA3A) and reelin (Yamashita et al., 2006); notably, the latter was an up-regulated gene in our wtPPT1 overexpressing cells (see Figure 7). Interestingly, CRMP1 (as well as other palmitoylated proteins) has been found dysregulated in *Ppt1*^−/−^ mice (Tikka et al., 2016; Segal-Salto et al., 2017), and it is an interacting partner of PPT1 (Scifo et al., 2015a).

NFs provide the structural support for elongation and stabilization of axonal processes during their maturation, maintainance of cytoskeletal organization and control radial axonal growth (Nixon and Shea, 1992; Lee and Cleveland, 1994; Letourneau, 1996; Elder et al., 1999; Lariviere and Julien, 2004). Down-regulation of mRNA encoding neurofilament proteins is a common feature of human neurodegenerative diseases (Julien and Mushynski, 1998), and mutated NF proteins can affect the dynamics of other cytoskeletal components of the axons, leading to fiber degeneration and neuronal death (Shea and Lee, 2013).

The reduced expression of GAP43, CRMP1 and both NF-H and NF-M genes and proteins in SH-p.wtCLN1 cells may account for the impaired axonal growth measured in our experimental setting (Supplementary Figure S7). Changes in the expression of genes encoding proteins of the synaptic compartment (neurotransmitter receptors, ion channels, membrane proteins) were also selectively found in SH-p.wtCLN1 (Figure 7). A positive correlation was found for the downregulated expression of SNAP25, a palmitoylated presynaptic protein, and of its related gene, in SH-p.wtCLN1 cells. SNAP25 is a plasma membrane protein of the pre-synaptic compartment, belonging to the SNARE complex involved in the exocytosis machinery (Wu et al., 2014). *SNAP25* gene polymorphisms are associated with ADHD, another neurodevelopmental disorder (Liu et al., 2017).

### Transcriptomic Profiling and NCL Genes Cross-Talk

Cross-talk among NCL genes and proteins has been identified and related to the pathogenesis of specific forms (Lyly et al., 2009; Chandra et al., 2015). Our transcriptomic analyses revealed up-regulation of mRNA *CTSD* in SH-wtCLN1 cells (see network in Figure 4B). *CTSD* encodes a palmitoylated lysosomal enzyme (Cathepsin D, CatD), involved in several biological processes (Koch et al., 2013; Khalkhali-Ellis and Hendrix, 2014). Mutations in *CTSD* are associated with congenital and infantile-onset NCL (MIM #610127; Siintola et al., 2006; Doccini et al., 2016). The processing of pro-CatD to mature CatD occurs in the lysosomal compartment, but its maturation is disrupted in the *Ppt1*^−/−^ mice, possibly due to the elevated lysosomal pH (Chandra et al., 2015; Bagh et al., 2017). Interestingly, CatD overexpression (both at the transcript and protein level) is linked to murine *Ppt1* knockout, though the cascade of events leading to enhanced regulation is yet unknown. Furthermore, an overexpression of PPT1 was recently detected in the brains of CLN4 patients, an adult NCL caused by mutations in DnaJ Heat Shock Protein Family (Hsp40) Member C5 (*DNAJC5*; MIM #162350; Nosková et al., 2011). The *DNAJC5* gene encodes a palmitoylated cytosolic protein (CSPα) associated with synaptic vesicles, possibly acting as substrate of PPT1 (Mastrogiacomo et al., 1994; Henderson et al., 2016). Mutations in the palmitoylated domain of CSPα are associated with accumulation of aggregated PPT1 by still unknown mechanism(s), and possibly altered PPT1 activity in different cell compartments (Henderson et al., 2016). However, no differential expression of *DNAJC5* mRNA was found in our RNA-seq surveys. Nevertheless, the biochemical link between the two diseases indicates that aberrant protein palmitoylation is crucial for neuronal degeneration, affecting the synaptic compartments in both conditions (Henderson et al., 2016).

### Concluding Remarks

The results of this study can be considered as the effect of a likely altered gene dosage of wt*CLN1* due to wild type gene overexpression. Altered gene dosage is considered as a molecular mechanism underpinning several human neurological conditions determined by either deletion or duplication of the same genetic traits (Lupski et al., 1991; Raskind et al., 1991; Chance et al., 1993; Woodward et al., 1998; Margolis et al., 2015). Although an overexpression and a loss-of-function models cannot be compared straightforwardly, our findings bring an evidence on the effects of dysregulated networks of genes coding for palmitoylated proteins in relation to the process of axonal sprouting and proliferation in this *CLN1*/PPT1- related experimental model. These findings are supported by a recent study on the role of palmitoylation in axonal dynamics (Holland and Thomas, 2017). An overexpression of the depalmitoylating enzyme may unbalance the fine tuning of the S-acylation process, thereby perturbing palmitoylated membrane proteins, which in turn may impact the CLN1 disease pathogenesis.

It is advised to consider that potential therapeutic strategies (gene therapy, enzyme replacement or others) would likely be more effective if they took into consideration the possible dysregulation of genes (and proteins) downstream to PPT1 (mal)function, including the disturbances in synaptic *palmitoylation networks*. In approaches to therapy, however, it is necessary to handle with caution the protein overexpression since the locoregional enhancement of a lysosomal enzyme may lead to cell stress, and hamper the predicted benefits of the therapy (Migani et al., 2017). We thus recommend that wide-range transcriptomic (as well as proteomic/metabolomics) investigations with a focus on affected molecular functions are carried out in animal models of genetic disorders prior to human interventions, if complementation therapies are considered as putative effective treatment of the underlying condition.

## Author Contributions

FP, FMS, MML, AS conceived and designed the study, wrote and drafted the manuscript; FP, MB, SB, FG, SD, RC performed transfection experiments, RNA-seq analysis, biochemical and morphological investigation; AD performed colocalization analysis; FP, MML performed bioinformatic analysis; AS, MML, FMS, MD checked the accuracy of data and the appropriateness of investigations; AS, MML gave the final approval of the manuscript for publication.

## Conflict of Interest Statement

AS received honoraria for consultancy from BioMarin Pharmaceutical, Inc. MML is a member of an Editorial board of Frontiers of Molecular Neuroscience. The other authors declare that the research was conducted in the absence of any commercial or financial relationships that could be construed as a potential conflict of interest.

## Footnotes

^1^http://www.ucl.ac.uk/ncl/mutation.shtml

^2^www.qiagen.com/ingenuity

^3^http://pantherdb.org

^4^http://www.cytoscape.org/

^5^http://swisspalm.epfl.ch/

^6^https://www.ncbi.nlm.nih.gov/geo/query/acc.cgi?token=srihcgwahfevtcd&acc=GSE98834

## Abbreviations

B-H *p-*value: Benjamini and Hochberg corrected *p*-value
CRMP1: Collapsin Response Mediator Protein 1
CTSD: Cathepsin D
DAPI: 4′,6-diamidino-2-phenylindole dihydrochloride
db-cAMP: Dibutyryl-cyclic AMP
DEG: Differentially Expressed Gene
DNAJC5: DnaJ Heat Shock Protein Family (Hsp40) Member C5
EA: Enzymatic Activity
EEG: Electroencephalogram
FE: Fold Enrichment
FDR: False Discovery Rate
FPKM: Fragments Per Kilobase per Million mapped reads
GAP43: Neuromodulin or Growth Associated Protein 43
IF: Immunofluorescence
IPA: QIAGEN’s Ingenuity Pathway Analysis^®^
LAMP2: Lysosomal Associated Membrane Protein 2
log2FC: Log2 fold change
LSD: Lysosomal Storage Disease
NCL: Neuronal Ceroid Lipofuscinosis
NFs: Neurofilament Proteins
PANTHER: Protein ANalysis THrough Evolutionary Relationships
PATs: Palmitoyl-Acyl Transferases
PPT1: Palmitoyl-Protein Thioesterase 1
qPCR: quantitative real-time PCR
RA: *all-trans* Retinoic Acid
RA-NBM: *all-trans* Retinoic Acid—Neurobasal medium
rhBDNF: recombinant human Brain-Derived Neurotrophic Factor
SEMA3A: Semaphorin 3A
SNAP25: Synaptosome Associated Protein 25
WB: Western blotting
zDHHC: Zinc finger S-acyl transferases.
