# Supplementary material for: The Networks of Genes Encoding Palmitoylated Proteins in Axonal and Synaptic Compartments Are Affected in PPT1 Overexpressing Neuronal-Like Cells
Source: Front Mol Neurosci. 2017 Aug 22;10:266. doi: 10.3389/fnmol.2017.00266 (PMC5572227; doi:10.3389/fnmol.2017.00266)
Supplement: Supplementary file 8 [file Supplementary_Material.pdf]

Supplementary Material

The networks of genes encoding palmitoylated proteins in axonal and synaptic compartments are affected in PPT1 overexpressing neuronal-like cells

Francesco Pezzini, Marzia Bianchi, Salvatore Benfatto, Francesca Griggio, Stefano Doccini, Rosalba Carrozzo, Arvydas Dapkunas, Massimo Delledonne, Filippo M Santorelli, Maciej Lalowski* and Alessandro Simonati*

*** Correspondence:** Corresponding Author: alessandro.simonati@univr.it
Co-corresponding Author: maciej.lalowski@helsinki.fi

1 Supplementary Methods

De-glycosylation assay

The removal of N-glycosylated residues was performed on 10 μg of protein homogenates in presence of PNGase F (Roche Diagnostics), overnight at 37°C. Untreated and de-glycosylated protein samples were resolved by SDS-PAGE under denaturing conditions. Proteins were transferred to PVDF membranes and probed with anti-PPT1 (Sigma Aldrich), as described in Material and Methods section. A prolonged electrophoretic run was performed to better resolve the PPT1 isoforms.

Morphometric evaluation of axonal-like structures

Differentiated and non-treated, engineered cell lines were analyzed using immunofluorescence of a SMI31-R antibody (Covance), which immunolabelled phosphorylated heavy chain neurofilaments (pNF-H) and, to a lesser extent, medium chain neurofilaments (pNF-M). Images were acquired at 10x magnification by Axiovision (Carl Zeiss); at least 1,500 cells were analysed. ImageJ and NeuronJ plugin were used to manually trace SMI31R immunolabelled structures. Both primary processes (sprouting from the cell body) and secondary processes (branching from a primary process) were traced. Only primary processes longer than 30 μm were taken into account for quantitative, comparative analysis. To normalize the number of neurofilament processes against the cellular density, nuclei were automatically quantified by CellProfiler 2.1.1. Data were collected from at least four independent experiments and reported as mean ± s.e.m; statistical analyses were performed using unpaired t-test versus empty-vector cells in the same culture conditions, considering as significant a p-value ≤0.05 and as highly significant a p-value ≤0.01.

2 Supplementary Figures

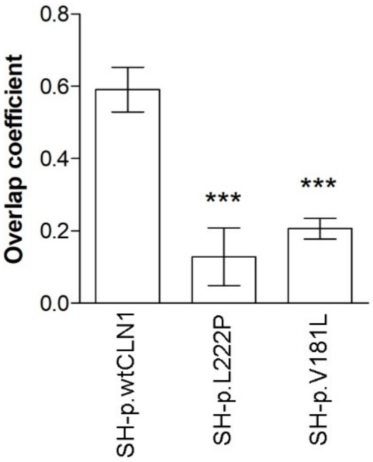

Supplementary Figure S1. Colocalisation analysis of PPT1/Lamp2 immunostainings of SH-SY5Y cell lines overexpressing a full-length PPT1.

The degree of colocalisation of PPT1 immunofluorescent signal on Lamp2 lysosomal staining was investigated in neuroblastoma cell lines which were transfected with either a wt*CLN1* or with cDNAs harbouring missense mutations (p.L222P and p.V181L). The quantitative analysis confirmed a reduced colocalization of mutated PPT1 in lysosomal compartment as compared to wtPPT1, in accordance with the diffuse immunostaining described in Figure 1 (pictures e, f, h and i). Mean ± s.d.; One-way ANOVA followed by Bonferroni's post-test; ***p<0.001.

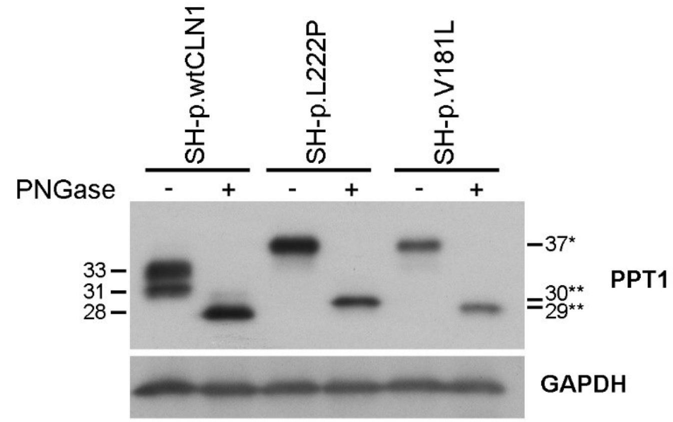

Supplementary Figure S2. Characterization of wild-type and missense mutation bearing SH-SY5Y PPT1 cell lines.

In protein homogenates isolated from wtPPT1 overexpressing cells, Western blot analysis with anti-PPT1 antibody revealed two bands of approximately 33 and 31 kDa, as detected in both parental (SH-SY5Y) and empty vector (SH-pcDNA3) cell lines. Following PNGase treatment to remove N-glycosylated residues, wtPPT1 migrated as a single band of approximately 28 kDa. Without PNGase treatment, both missense mutation-bearing cell lines (SH-p.L222P and SH-p.V181L) exhibited a band running at higher molecular weight (37 kDa). Upon PNGase treatment, the same cells exhibited a shift in migration of ~1-2 kDa in comparison to de-glycosylated wtPPT1 (marked by asterisks). "-" not treated sample, "+" PNGase treated samples. In this experimental setup PPT1 proteoforms migrated at molecular weights lower than reported in Figure 1, due to conditions of SDS-PAGE, allowing for better resolving each of the PPT1 forms.

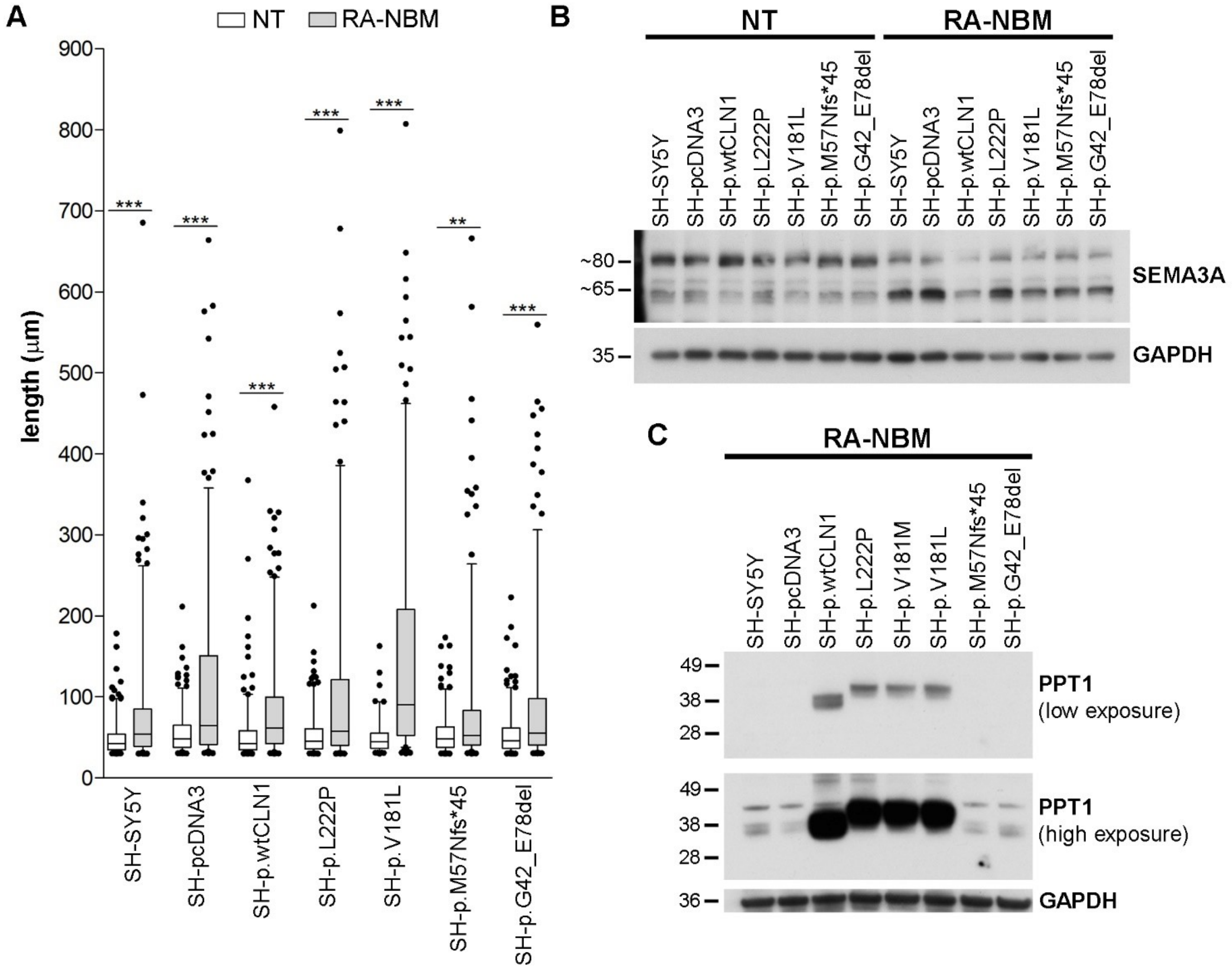

Supplementary Figure S3. Neuronal differentiation of *CLN1* transfected cell lines following exposure to RA-NBM media.

(A) Morphometric evaluation of phosphorylated NF-H immunostained processes demonstrated the efficacy of RA-NBM treatment to induce a significant elongation of axonal-like structures in all *CLN1* transfected cell lines. An equal amount of processes was evaluated in three independent experiments (n=230). Boxes and bars enclosed the 5° and 95° percentiles, whereas horizontal black lines represent the medians. Statistical analysis was performed by One-way ANOVA followed by Bonferroni's multiple comparison test on NT (not treated) and RA-NBM conditions for each cell lines; ** $p<0.01$, *** $p<0.001$. **(B)** Semaphorin 3A conversion from the 80 kDa to 65 kDa isoform was observed in all *CLN1* transfected SH-SY5Y cell lines following differentiation in RA-NBM media, as previously reported on parental SH-SY5Y cell line (Pezzini et al., 2017). **(C)** Immunoblotting analysis at the end of the differentiation paradigm (9 days *in vitro*) confirmed that the protracted growth in RA-NBM medium did not affect the expression of PPT1.

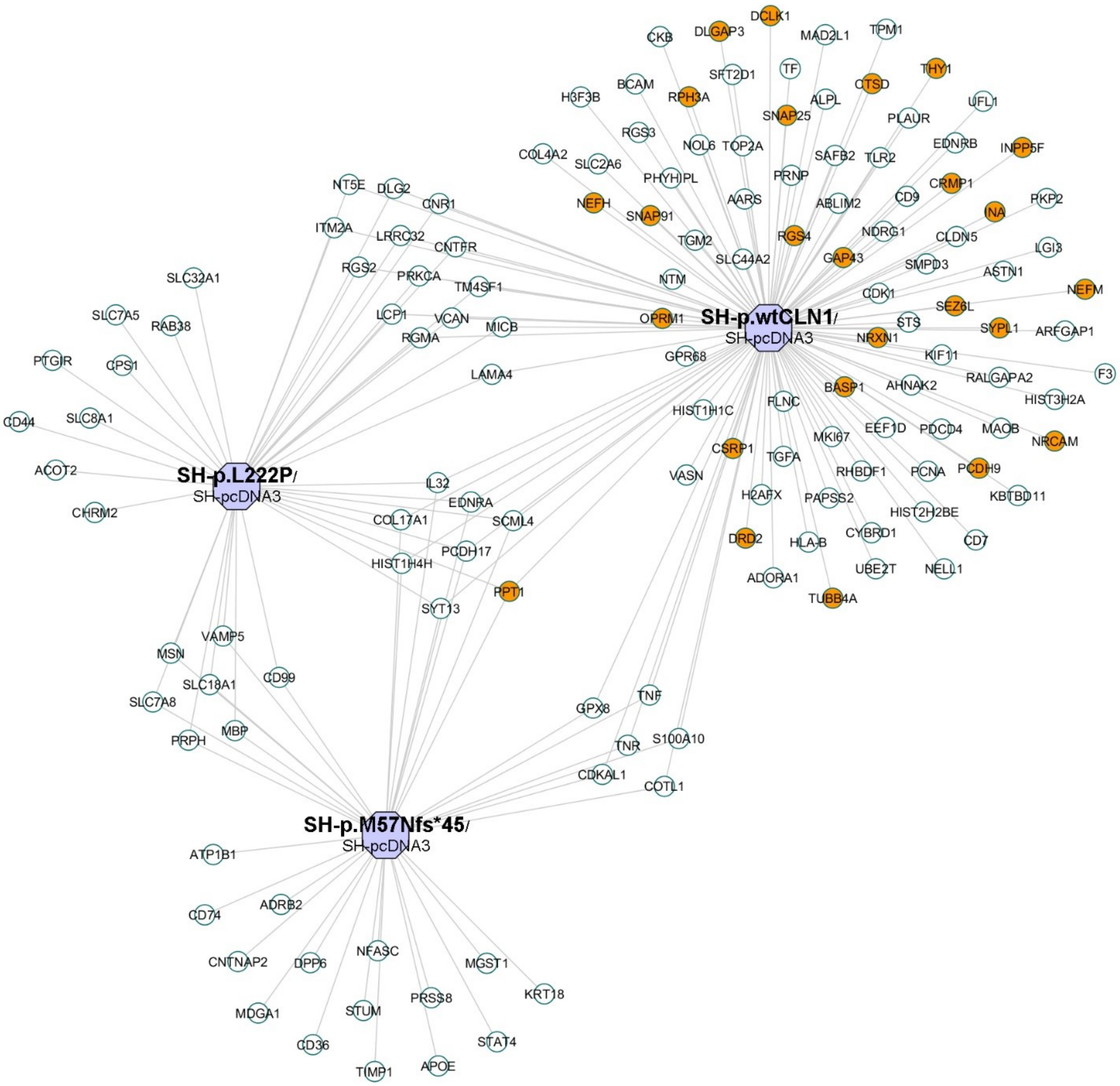

Supplementary Figure S4. Network of palmitoylated protein encoding genes identified in the transcriptomic profiles of *CLN1* transfected cell lines.

Network depicting the subsets of genes embedded in intersections of a Venn diagram from Figure 4A. Twenty-five genes, specifically expressed in SH-p.wtCLN1 (except PPT1), and linked to IPA attributes and GO terms in Figure 4B are portrayed in orange.

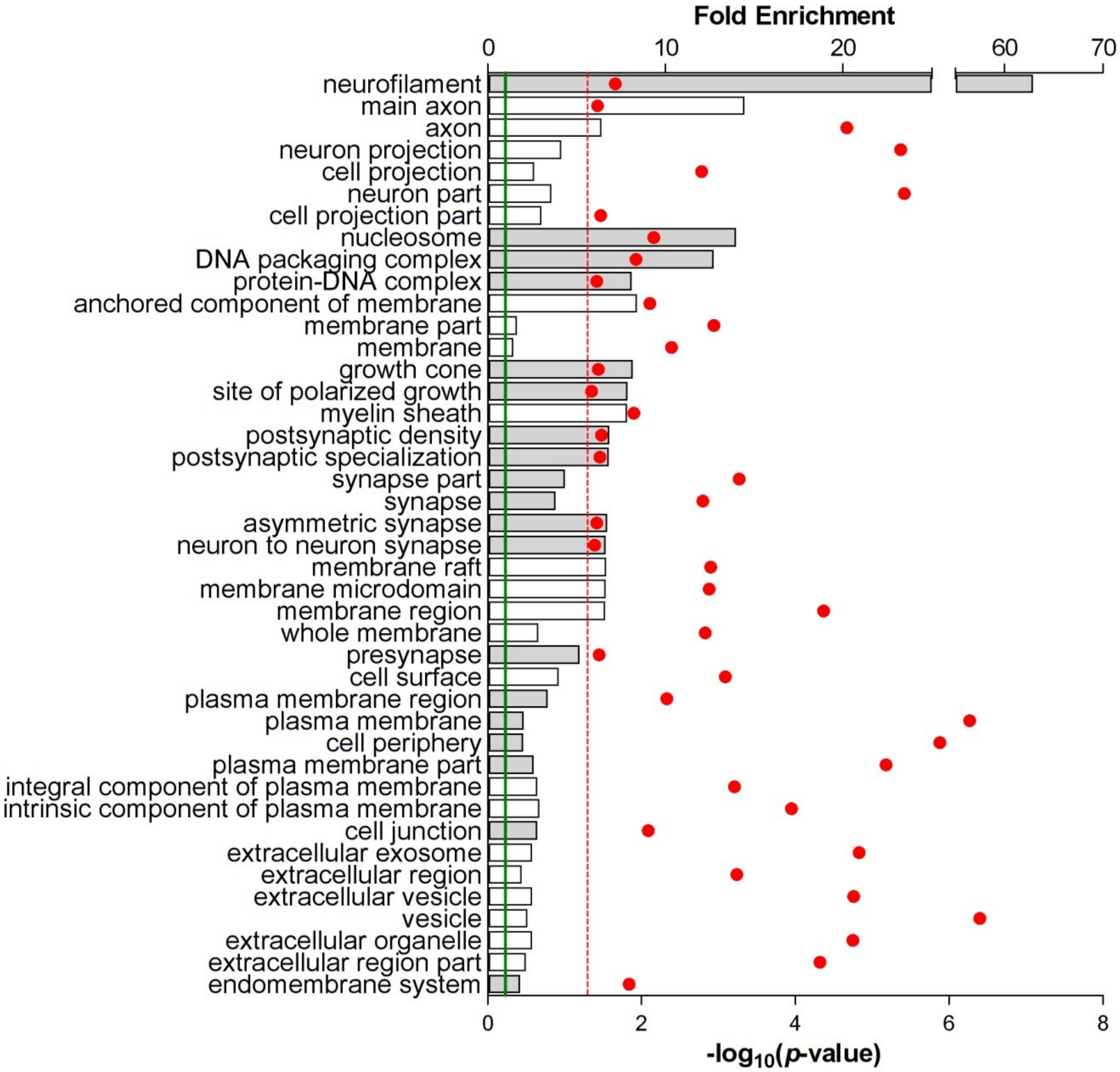

Supplementary Figure S5. Cell Component GO term enrichment analysis of palmitoylated protein encoding genes identified in SH-p.wtCLN1 cells.

Several GO terms referred to neuronal components of membrane processes (*neuron projection*, *growth cone* and *axon*) as well as to synaptic compartments (*synapse*, *presynapse*, *postsynaptic specialization*). Fold enrichment (bar) represents the number of input genes observed against the expected number of genes assigned to a specific GO term; a fold enrichment >1 (green line) indicates that the category is overrepresented in the given experimental setting. *P*-value (red dots), reported as $-\log_{10}$, is determined by the binomial statistic and represents the probability that the number of genes observed in a category occurred by chance; the threshold is set to 1.3 (red line), which corresponds to a *p*-value <0.05.

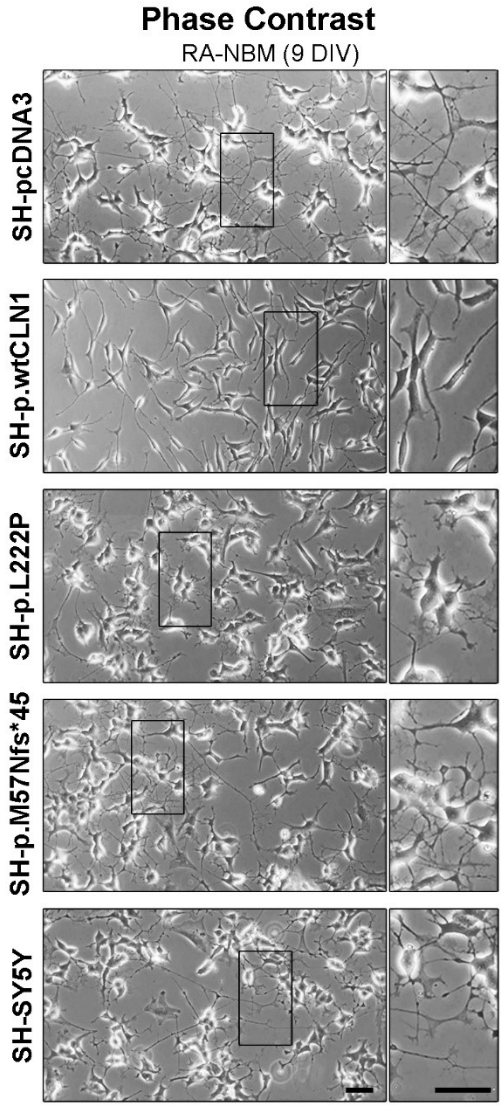

Supplementary Figure S6. Qualitative morphological features of *CLN1* cell lines following neuronal differentiation in RA-NBM medium.

Morphological changes in *CLN1* transfected cells lines were checked by phase contrast microscopy during neuronal differentiation. Exposure to RA-NBM medium induced a significant elongation of membrane processes in all cell lines, with more arborisations at the end of differentiation (9 DIV). SH-p.wtCLN1 cells showed less arborized and stunted processes in comparison to mock-transfected cells and other *CLN1* transfected cell lines. DIV, days *in vitro*; scale bar equals to 50 µm.

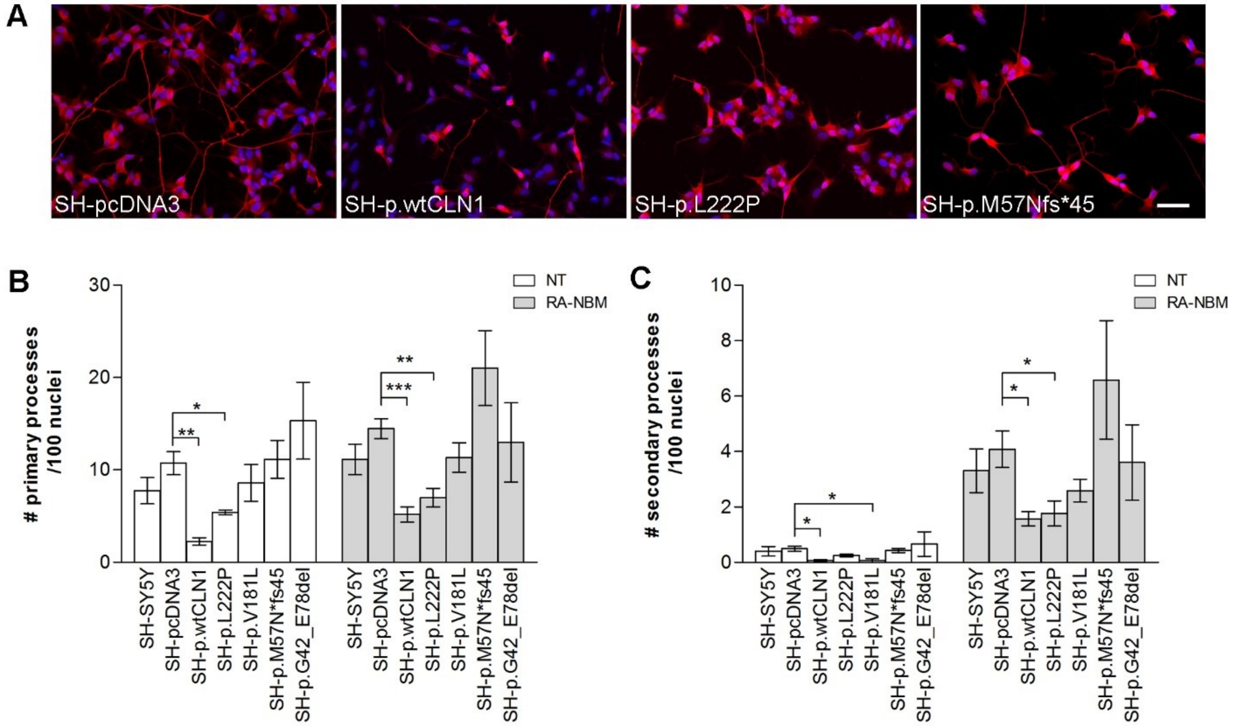

Supplementary Figure S7. Morphometric evaluation of neurofilament immunolabelled structures following neuronal differentiation in RA-NBM medium.

(A) Axonal-like structures were evaluated following immunohistochemistry for phosphorylated heavy chain neurofilament pNF-H (by SMI31R antibody). Elongated and ramified processes could be observed in parental and SH-pcDNA3 cells; conversely SH-p.wtCLN1 exhibited short, stunted branches. Several short branches were detectable in SH-p.L222P whereas nonsense mutation-bearing cells demonstrated numerous processes of different length, similar as in the controls. Nuclei are marked in blue; scale bar equals to 50 µm. **(B-C)** Morphometric evaluation confirmed a significant reduction in density of pNF-H immunolabelled primary processes (B), as well as secondary processes (C) in wt*CLN1* transfected cells, both under basal conditions (white bars) and following RA-NBM differentiation (grey bars). Similarly, SH-p.L222P clone displayed a reduced density of primary processes in both culture conditions, whereas a significant reduction in density of secondary processes was seen for the two missense mutated cells in one culture condition only (NT for SH-p.V181L and RA-NBM for SH-p.L222P). The morphometric evaluation of indel mutation-bearing cells was associated with a much higher variability. Mean ± s.e.m.; unpaired *t*-test versus empty-vector cells (SH-pcDNA3); * $p<0.05$, ** $p<0.01$, *** $p<0.001$; NT- not treated; RA-NBM- differentiated cells.
